# Temperature-Responsive Transmission Switching in Smart Glass Comprising a Biphasic Liquid Crystal

**DOI:** 10.3390/ma18214989

**Published:** 2025-10-31

**Authors:** Min-Han Lu, Yu-Cheng Chiang, Wei Lee

**Affiliations:** 1 Institute of Lighting and Energy Photonics, College of Photonics, National Yang Ming Chiao Tung University, Guiren Dist., Tainan 711010, Taiwan; p10061203@gmail.com; 2 Institute of Photonic System, College of Photonics, National Yang Ming Chiao Tung University, Guiren Dist., Tainan 711010, Taiwan; willjames770627@gmail.com; 3 Institute of Imaging and Biomedical Photonics, College of Photonics, National Yang Ming Chiao Tung University, Guiren Dist., Tainan 711010, Taiwan

**Keywords:** biphasic liquid crystals, smectic A*, chiral nematic, high haze, high contrast ratio

## Abstract

This study investigates the temperature-driven transmission switching behavior of our proposed smart glass, which utilizes a biphasic liquid crystal system under continuous application of a distinctive homeotropic (H) state voltage (*V*_H_). By ascertaining *V*_H_ at temperatures near the phase transition point, the minimum voltage required to sustain the H state in the smectic A* (SmA*) phase is identified. Interestingly, this minimum *V*_H_ is unable to induce the H state in the chiral nematic (N*) phase, thereby maintaining a low-transmission scattering state; i.e., the focal conic (FC) state. This empowers passive, bidirectional optical switching between the transparent H state (in the SmA* phase) and the scattering FC state (in the N* phase) in an unaligned liquid crystal cell. This work employs two dissimilar chiral dopants, R811/S811 and CB7CB/R5011, both capable of inducing the SmA* phase. Neither resulting cell system underwent surface orientation treatment, and a black dye was incorporated to enhance the contrast ratio. The results indicate that the more efficacious CB7CB/R5011 system achieves a contrast ratio of 17 between the transparent and scattering states, with a corresponding haze level of 78%. To further reduce energy consumption, the experimental framework was transitioned from a continuous-voltage to a variable-voltage mode, giving rise to an increased haze level of 88%. The proposed switching scheme holds promise for diverse applications, notably in smart windows and light shutters.

## 1. Introduction

Advancements in science and technology have broadened the application of liquid crystals (LCs) beyond their conventional role in displays [1], with recent years witnessing growing interest in their potential for energy-saving components. Consequently, numerous low-power-consumption LC devices with energy-efficient functionality have emerged. When it comes to LC energy-saving devices, smart windows [2,3] stand out as a prominent example. Its multifunctional capabilities make it a potential solution for various lifestyle applications, including peep-proof [4], light baffles, and energy conservation. The primary characteristic of smart windows is their ability to transition between distinct optical states in response to varying requirements. Typically, they can be maintained in the homeotropic (H) state with high transmittance and clarity, and switched to the scattering state [5,6,7] when external light becomes excessive or when gentle lighting is necessary. A substantial body of research has been conducted on LC smart windows, encompassing a diverse range of materials and techniques, including doped polymers, ionic additives, photosensitive materials, and specialized electrodes [8,9,10,11]. These developments demonstrate significant market potential for commercial applications and are consequently attracting growing attention. Additionally, cholesteric liquid crystals (CLCs) hold considerable promise for application owing to their unique structural properties, which have garnered substantial interest from researchers in this field. The helical structure and Bragg-reflection characteristics of the Grandjean planar (P) state [12,13] allow a special design for the reflection of near-infrared wavelengths of light, thereby achieving the effect of heat insulation [14,15]. The majority of the mechanisms utilized in CLC smart windows permit high transparency in the H state and low transmission in the focal conic (FC) state, enabling reversible switching between the two configurations [16,17].

Yoon et al. proposed a method by incorporating an ionic additive and a black dye into CLC containing polymer monomers [18]. These components were mixed and injected into an uncoordinated LC cell. Subsequently, a voltage was applied to align the LC and dye molecules in the H state. In addition, ultraviolet (UV) light was used to cure the monomers to sustain the initial state of the sample in a high-transmission H texture. Furthermore, a direct-current (DC) voltage was applied to induce the disturbance of ions, generating the electrohydrodynamic flow that tilts the LC molecules. Upon the removal of the voltage, optically stable FC state was achieved. This process enables the switching between the H and FC states. Nevertheless, the polymer structure inherent to this architectural configuration results in a required high driving voltage and a complex fabrication process. Moreover, the dopant ions often influence the device’s operational lifetime. Immediately following this procedure, the same group employed LCs doped with a black dye and injected into a mixed-alignment cell to switch the samples between the high-transmission state in the smectic A (SmA) phase, the intermediate-transmission state in the N phase, and the low-transmission isotropic state by varying the temperature (*T*). The group also applied the mixed-alignment technique to modify the pretilt angle. In the SmA phase, the black dye and LC molecules were vertically aligned, exhibiting high transparency owing to the LC characteristics of the SmA texture. As *T* rose and the sample transitioned to N phase, the LC and dichroic-dye molecules were oriented at an angle along the rubbing direction. The dye molecules then absorb linearly polarized light along the same direction, leading to a decrease in transmittance. Upon entering the isotropic phase, the LC and dye molecules are arranged in a disordered manner, resulting in the lowest-transmission state. To sum up, within this framework, when *T* is low, the sample demonstrates high-transmission state, allowing sunlight to enter the room. Conversely, as *T* rises, transmission decreases, reducing the amount of light entering the room and subsequently lowering the room *T*. While this mechanism is capable of automatically adjusting transmission, it lacks the scattering state, which is necessary to achieve the functions of peep-proof and shading [4]. In the same year Yoon et al. presented another relative work, in which LC was impregnated with an azobenzene and a black dye and the mixture was injected into vertical-alignment LC cells. The LC is initially in the highly transmissive H state imposed by the vertical alignment layers. As *T* rises to the phase transition point, it undergoes a change from the (low-transmission) nematic (N) phase to the (high-transmission) isotropic state. In the latter state, the black dye as a dopant causes the sample to appear in a low-transparency state with a low haze. If the sample is exposed to UV light when it is initially in the N phase, the clearing point *T*_c_ will drop due to photoisomerization of the azo molecules from *trans* to *cis* [19,20,21]. This leads to a transition of the LC from the high-transmission N phase to the low-transmission isotropic phase. However, a disadvantage of this switching principle between high- and low-transmission states is that a thicker LC cell (20 μm) is required. Furthermore, this mechanism lacks scattering states, which precludes the achievement of the function of peeping prevention [14].

The present study makes use of two distinct phases, namely the smectic (SmA*) and chiral nematic (N*) phases, in an unaligned LC cell, with the objective of achieving a reversible switching process between the transparent and scattering states [22,23]. Two chiral systems were utilized. The first system employed a binary chiral dopant (R811/S811), which has been shown to induce the smectic phase [15,24,25]. The second system involved the mixing of a bent-core LC dimer with a chiral agent (CB7CB/R5011) as a dopant to bring about the smectic phase of a LC [26]. A trace amount of dichroic black dye was dispersed into both systems, and the subsequent changes in their electro-optical characteristic were observed and documented. The CB7CB/R5011 system incorporating the black dye achieved an optimal contrast ratio (CR) of approximately 15–17, in contrast to the dye-free counterpart, which exhibited a CR of only 7–10, representing a two-fold enhancement. Furthermore, modifications have been implemented by substituting the power-consuming continuous-voltage mode with a variable-voltage alternative. By harnessing the SmA*–N* phase transition in biphasic LCs, the proposed technology offers notable advantages—high CR, energy efficiency, and versatile operation. Its precise light control with minimal power makes it ideal for smart windows, automotive glazing for thermal and glare control, and advanced optical devices (such as light shutters and adjustable filters)—contributing significantly to energy savings and carbon neutrality.

## 2. Materials and Methods

### 2.1. Materials

This research was classified according to the materials employed, with two principal additives identified, the R811/S811 system and the CB7CB/R5011 system. Firstly, in the R811/S811 system, the host employed is the calamitic nematic LC E7 (Daily Polymer Corp., Kaohsiung, Taiwan). E7 with a rod-like molecular shape is a cyano-based and four-component eutectic mixture, with dielectric anisotropy Δ*ε* of +14.3 (measured at frequency *f* of 1 kHz and *T* = 20 °C), birefringence Δ*n* of 0.2255 (at wavelength *λ* of 589 nm and *T* = 25 °C), and *T*_c_ = 59 °C. The composition was functionalized by incorporating the left-handed chiral additive S811 (10 wt.%) and its right-handed counterpart R811 (20 wt.%). Both chiral agents were sourced from Yung Zip Chemical Industry Corp., Taichung, Taiwan. The helical twisting powers (HTPs) of the chiral dopants R811 and S811 in E7 are 11 μm^−1^ and −11 μm^−1^, respectively. This concentration ratio follows the demonstration by Tsay et al. for inducing the SmA phase in E7 [25], and is further supported by our team’s previously published journal papers on the subject [15,24,25]. Secondly, in the CB7CB/R5011 system, we doped CB7CB (1″,7″-bis(4-cyanobiphenyl-4′-yl)heptane), synthesized by Jiangsu Hecheng Display Technology Corp., Nanjing, China, with *T*_c_ = 116 °C. CB7CB is a bent mesogenic dimer known to exhibit a nematic twist-bend phase (N_TB_) below 99 °C [27]. This phase is characterized by a high flexoelectric coefficient of approximately 31 pC·m^−1^ and low Δ*ε* around +1 and +2 [28]. Subsequently, the right-handed chiral additive R5011 (1 wt.%) was introduced into the LC mixture to form a CLC. R5011, supplied by Merck, Darmstadt, Germany, demonstrates a HTP of 115 μm^−1^ in E7.

**Figure 1:**
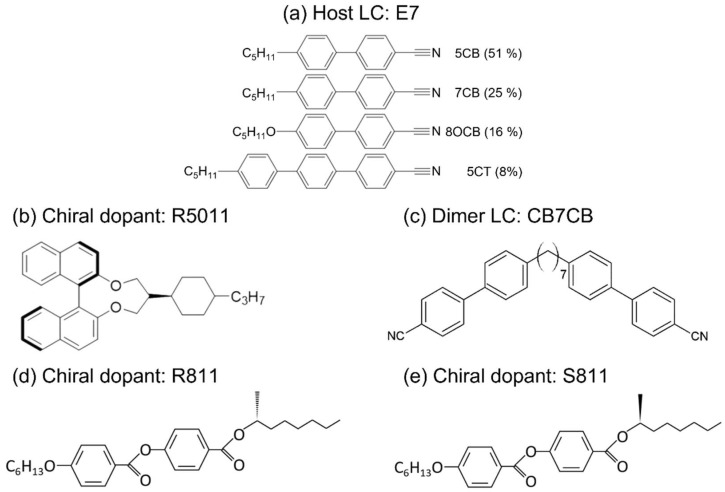
Schematic of the chemical structures of (**a**) E7, (**b**) R5011, (**c**) CB7CB, (**d**) R811, and (**e**) S811.

Furthermore, the dichroic black dye S428 [4], produced by Mitsui, Tokyo, Japan, was added to both the original R811/S811 and CB7CB/R5011 systems to enhance CR. The chemical structures of all aforementioned materials are illustrated in Figure 1, and were used without further purification.

The prepared CLC cells were categorized into two primary groups for differentiation. Initially, they were divided according to the distinct material systems—R811/S811 and CB7CB/R5011—as shown in Table 1 and Table 2. Within each system, further classification was made into two subgroups based on the presence or absence of black dye doping. The compositions of the experimental samples for the R811/S811 and CB7CB/R5011 systems are summarized in Table 1 and Table 2, respectively. In the R811/S811 system, the total concentration of chiral additives is fixed at 30% with an R811:S811 ratio of 20:10 wt.%. This partial racemic modification leaves a small positive net helical twisting power and shifts the SmA*–N* transition temperature toward room temperature, thereby improving the practical applicability of the CLC smart window in daily life. The 10 wt.% excess of the right-handed dopant (R811) produces a net HTP comparable to that provided by 1 wt.% R5011 in the other system (CB7CB/R5011 system), enabling meaningful cross-system comparisons at similar HTP scales. In the CB7CB/R5011 system, the host composition E7:CB7CB = 50:50 wt.% was chosen based on prior observations that substantial CB7CB addition to E7 can induce a SmA phase [26]. Our screening confirmed that this blend places the SmA*–N* transition temperature near room temperature. A small addition of the right-handed dopant R5011 (1 wt.%), which exhibits high HTP, establishes a robust cholesteric bias in the 50 wt.%-to-50 wt.% ratio of E7 to CB7CB host.

### 2.2. Sample Preparations

LC mixtures were prepared using a high-precision microbalance (Mettler Toledo AX26, Mettler Toledo, Gießen, Germany) to accurately configure the component concentrations required for the experiment samples. Subsequently, each prepared mixture was transferred into a glass sample vial placed on a heating platform (IKAC-MAG HS7, IKA-Werke GmbH & Co. KG, Staufen, Germany). The temperature was maintained at 120 °C for 10 min to drive the LC mixture into the isotropic phase, ensuring thorough homogenization via magnetic stirring.

Empty cells were fabricated from two patterned indium–tin-oxide (ITO) glass substrates (supplied by Chiptek Technology Co., Ltd., Miaoli, Taiwan) without any surface orientation treatment. The cell assembly process was as follows: an epoxy AB adhesive, premixed with either 5.5 μm or 15 μm spacers, was applied along the edges of the upper and lower ITO substrates to bond them together and define cell gap with an overlapping electrode area of 0.25 cm^2^. Electrical connections were established by soldering wires to the electrode regions on the non-overlapping edges of the substrates. Following cell fabrication, both the LC mixture and the cell were placed on the heating platform at 120 °C. An appropriate volume of the LC mixture was drawn using a pipette and introduced into the LC cell via capillary action. Upon complete filling, the cell was removed from the heating platform and allowed to cool naturally to room *T* over 1 h, thereby completing the sample preparation process.

**Figure 2:**
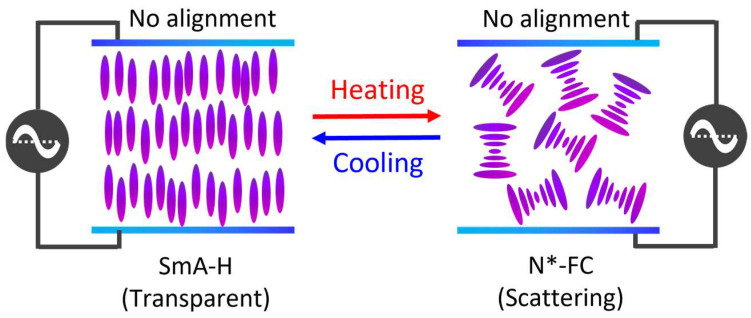
Mechanism of the experimental framework for continuous-voltage mode application.

The aim of this study is to investigate and demonstrate the ability of the LC cell to reversibly switch between H and FC states, enabling reversible transition between the transparent and scattering states. Initially, a continuous alternating-current (AC) voltage was applied across the cell to induce the high-transmission SmA*–H state. Thereafter, the LC cell was heated while maintaining the applied voltage. Upon reaching the SmA*–N* phase transition *T*, with the voltage conditions remaining unaltered, the cell transitions from the transparent H state of the SmA* phase to the scattering FC state of the N* phase, rather than to the transparent H state of the N* phase. This behavior arises because the applied voltage amplitude is sufficient to sustain the SmA* phase but insufficient to induce the H state in the N* phase. Cooling the cell below the SmA*–N* transition *T* under identical voltage conditions restores the H state of the SmA* phase. This mechanism enables passive, bidirectional switching between transparent and scattering states. The operational principle is illustrated in Figure 2.

### 2.3. Dielectric Measurement

The phase transition sequence and dielectric data of the samples were measured using a high-precision LCR meter (Agilent E4980A, Agilent Technologies, Santa Clara, CA, USA) [29]. The probe voltage was set to 0.5 V_rms_ for frequency scans spanning from 20 Hz to 2 MHz. The temperature range was set to 85 °C to 15 °C using a precision temperature controller (Linkam T95-PE, Linkam Scientific Instruments, Salfords, UK) with a cooling rate of 0.5 °C·min^−1^. Ultimately, a LabVIEW program was employed for the configuration and operation of the LCR meter and the precision *T* controller. The experimental setup allows for the measurement of the capacitance (*C*_P_) and conductivity (*G*_P_) of the samples at different *T*. Subsequently, the measured *C*_P_ and *G*_P_ are incorporated into Equations (1) and (2) to ascertain the real ($\varepsilon^{\prime }$) and imaginary ($\varepsilon^{\prime \prime }$) pasts of the dielectric constant of the sample under examination in accordance with:

(1) $$\varepsilon^{\prime }(\omega )=\frac{C_{P}}{C_{0}}=\frac{C_{P}d}{\varepsilon_{0}A},$$

(2) $$\varepsilon^{\prime \prime }(\omega )=\frac{1}{\omega C_{0}R_{P}}=\frac{G_{P}d}{\omega \varepsilon_{0}A},$$

and

(3) $$\varepsilon *\;=\varepsilon ’-i\varepsilon^{\prime \prime },$$

where *C*_0_ represents the capacitance of the empty cell, $\varepsilon_{0}$ denotes the vacuum dielectric constant, *A* is the electrode area, *ω* is the angular frequency, *d* is the cell gap of the LC cell, and *R*_P_ is the measured resistivity. Furthermore, by utilizing the values of $\varepsilon^{\prime }$ and $\varepsilon^{\prime \prime }$, one can derive the complex dielectric constant ε* from Equation (3). Once the dielectric spectra were obtained at each *T*, the value at *f* = 10 kHz was extracted from each spectrum. The data were then analyzed using appropriate software, and the resulting curve was plotted to show the change in $\varepsilon^{\prime }$ with *T*. A pronounced change in the curve indicates a distinct phase transition at a specific *T*. To better resolve these transitions, the first-order derivative with respect to *T* was calculated, and the resulting extrema identified the temperature at which $\varepsilon^{\prime }$ underwent the most significant change, namely the phase transition point.

### 2.4. Measurement of Optical and Electrooptical Characteristics

#### 2.4.1. Optical Texture Observation

To observe phase transitions and corresponding optical textures of the SmA* and N* phases, the samples were placed on a precision *T* controller (Linkam T95-PE). *T* was adjusted to induce specific phase changes. An AC square-wave voltage was applied to the samples using an arbitrary function generator (Tektronix AFG-320, Tektronix, Inc., Beaverton, OR, USA) coupled with a signal amplifier (Trek Model 603, Advanced Energy Industries, Inc., Fort Collins, CO, USA). Optical textures were examined under a polarizing optical microscope (POM, Olympus BX51-P, Olympus Corporation, Tokyo, Japan) in transmission mode with a 20× objective lens and crossed polarizers. Images were captured with a digital camera (Olympus XC30, Tokyo, Japan) and transferred to a computer for observation and documentation. The recorded textures were then compared with the known phase transition sequence to verify the corresponding transition temperature.

#### 2.4.2. Electrooptical Properties

A laser-based optical measurement system was employed to characterize the voltage−transmittance (*V*–*T*%) response and determine the homotropic state voltage *V*_H_ of the LC cell under various phases and voltage conditions. The *T* controller (Linkam T95-PE) was positioned in the optical path, with the LC cell mounted onto its stage. The AC electric field applied to the cell via a LabVIEW-controlled signal generator (Tektronix AFG-320), amplified by a signal amplifier (Trek Model 603). A He–Ne laser (JDSU 1202-1, JDS Uniphase Corporation (JDSU), Milpitas, CA, USA) served as the light source, passing through a neutral-density filter before illuminating the cell. Transmitted light was detected by a photodetector and analyzed by computer to evaluate the relationship between transmittance intensity and applied voltage (amplitude and frequency).

#### 2.4.3. Transmission Spectra Measurement

Visible-light transmission spectra were measured to investigate transmittance variations as a function of *T* and phase transition sequence. The sample was mounted on another Linkam T95-PE *T* controller. An AC electric field was applied using a signal generator (Tektronix AFG-320) and amplified by a signal amplifier (Trek Model 603). The *T* controller was positioned between a halogen light source (Ocean Optics HL2000, Ocean Optics, Inc., Orlando, FL, USA) and a fiber-optic spectrometer (Ocean Optics HR2000+, Ocean Optics, Inc., Orlando, FL, USA). Light from the halogen source was directed perpendicularly onto the sample via an optical fiber, and the transmitted signal was collected by the spectrometer and analyzed over the wavelength range of 400–800 nm. Transmittance variations were further correlated with POM texture observations to confirm the phase transition sequence.

#### 2.4.4. Haze Measurement

The low-transmittance mechanism employed in our system is based on the scattering state. Therefore, transmittance alone is insufficient to evaluate the performance of the smart window when functioning solely as a scattering element. To address this limitation, haze measurements are required. Measurements were conducted using a haze meter (COH-5500, Nippon Denshoku Corporation, Tokyo, Japan), a precision TEC controller (WISE LIFE CDS15008RRA, WISE LIFE TECHNOLOGY CO., LTD., Keelung City, Taiwan), a signal generator (Tektronix AFG-320), and a signal amplifier (Trek Model 603). These instruments enabled the generation of disparate phases and optical textures by modulating *T* and voltage, allowing for corresponding haze characterization.

The haze meter is equipped with a calibrated white LED light source and an integrating sphere featuring a detection aperture of 0.5 × 0.5 cm^2^. When light traverses a medium, it interacts with impurities, particles, surface irregularities, and other forms of non-uniformity, resulting in scattering and increased haze. This scattering causes portions of light to deviate from their original propagation path, dispersing in various directions.

To measure haze, the LC cell was situated in the optical path with its glass substrates oriented perpendicular to the incident light. The fraction of diffuse light—defined as light dispersed by more than 2.5°—relative to the total transmitted light was measured across the 400–700 nm wavelength range using the integrating sphere, and calibrated against the instrument’s scattering baseline.

Haze is expressed as a percentage, calculated by the ratio of diffuse light intensity *I*_d_ to total transmitted light intensity *I*_t_, as defined by the following equation:

(4) $$\mathrm{Haze}\;(\%)=\frac{I_{d}}{I_{t}}\times 100\%.$$

## 3. Results and Discussions

This study investigated the switching behavior between the SmA*–H state and the N*-FC state, focusing on their influence on transparent (SmA*–H) and scattering (N*-FC) states. All LC cells used were prepared without any surface orientation treatment. Initially, a continuous AC voltage was applied to the LC cell in the SmA* phase to maintain the H state with high transmittance. When the cell was heated to the SmA*–N* phase transition *T* under constant voltage, the optical state shifted from transparent SmA*–H to scattering N*-FC, rather than proceeding to the N*-H state. Upon cooling below the SmA*–N* transition *T* under identical voltage conditions, the system reverted to the SmA*–H state. As illustrated in Figure 2, this mechanism facilitates passive, bidirectional transitions between two contrasting configurations. We explored two material systems: CLC samples with R811/S811 (Table 1) and CB7CB/R5011 (Table 2). To examine the effect of a black dye additive, each system was supplemented with a variant containing dichroic black dye, S428, designated as S1-2 and S2-2. The optical and electro-optical properties of both systems were systematically characterized, and the experimental configurations were optimized accordingly.

### 3.1. Birefringence in the LC Material E7

**Figure 3:**
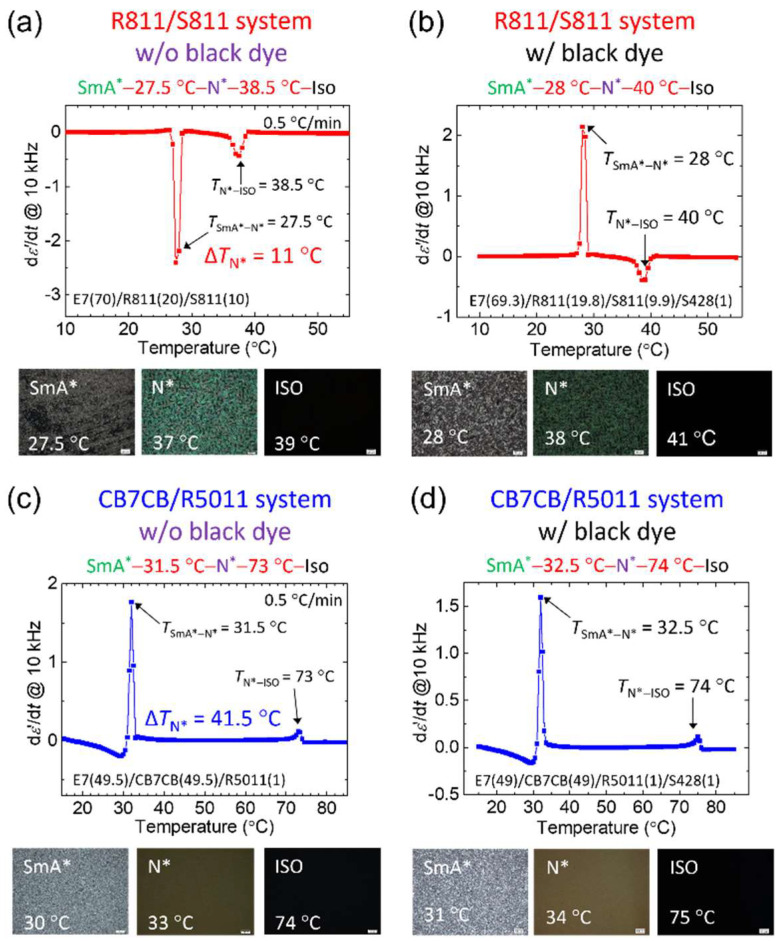
*T*-dependent first derivative of $\varepsilon^{\prime }$ with respect to *T* and optical textures at different *T* in the (**a**) R811/S811 system without black dye S428 and (**b**) with S428; in the (**c**) CB7CB/R5011 system without S428 and (**d**) with S428. The nematic host of these CLC samples is E7. Scale bar: 200 μm.

Since the modulation between high- and low-transmission states in this work relies on the SmA*–N* phase transition, phase sequence identification was initially performed using an LCR meter combined with polarizing optical microscopy to determine the operating *T* ranges of both R811/S811 and CB7CB/R5011 systems. All measurements were conducted under controlled cooling conditions, with *T* decreased at a rate of 0.5 °C·min^−1^. The R811/S811 system was first evaluated under two conditions—without and with the addition of black dye—across a temperature range from 55 °C to 10 °C. Based on the first-order derivative of the dielectric spectra with respect to *T*, complemented by corresponding optical textures, the phase sequences were identified. As revealed in Figure 3a,b, the SmA*–N* transition *T* for the dye-free and dye-doped samples were 27.5 °C and 28 °C, respectively, while the N*–Iso phase transitions occurred at 38.5 °C and 40 °C, respectively. Notably, the SmA*–N* occurred near room *T*, and the temperature span of the N* phase (Δ*T*_N*_) was approximately 11 °C for the dye-free CLC, representing the tunable range of the low-transparency state. As evidenced by the results in Figure 3a,b, the incorporation of black dye has a negligible effect on the phase transition *T*.

Similarly, the phase transitions of the CB7CB/R5011 system were measured over a temperature range from 85 °C to 15 °C. As shown in Figure 3c,d, the phase sequences of the undoped and black dye-doped samples are presented alongside their corresponding optical textures. The SmA*–N* phase transition *T* were 31.5 °C (undoped) and 32.5 °C (dye-doped), while the N*–Iso transitions occurred at 74 °C and 73 °C, respectively. Δ*T*_N*_ was 41.5 °C for the dye-free sample. These results are consistent with those of the R811/S811 system, confirming that the presence of black dye has minimal influence on phase transition *T*. The SmA*–N* transitions in both systems occurred near room *T*; nonetheless, Δ*T*_N*_ of the CB7CB/R5011 (41.5 °C) significantly exceeded that of the R811/S811 system (11 °C). indicating a broader tunable range in the scattering state and improved *T* control capability.

**Figure 4:**
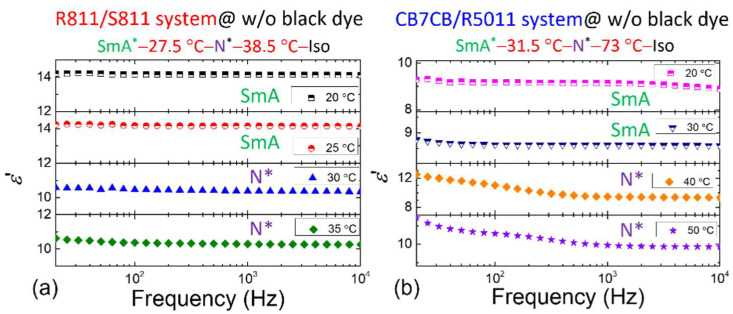
$\varepsilon^{\prime }$ spectra of the dye-free (**a**) R811/S811 and (**b**) CB7CB/R5011 CLC systems at various *T* with the phase transition sequence of each CLC system shown above. The nematic host of these CLC samples is E7.

While measuring the phase transition *T*, dielectric spectroscopy was also conducted for both systems undoped with black dye. The dielectric properties of the R811/S811 system in the SmA* and N* phases are depicted in Figure 4a. Noticeably, within the measured frequency range, both phases exhibit nearly constant permittivity, with no discernible dielectric relaxation. In contrast, Figure 4b presents the results for the CB7CB/R5011 system, where the SmA* phase similarly shows no dielectric relaxation, but the N* phase displays pronounced dielectric relaxation behavior. Comparative analysis reveals that such relaxation behavior is exclusive to the CB7CB/R5011 system at elevated *T*, suggesting it is a distinctive feature of CB7CB’s flexoelectric polarization [28,30,31]. Moreover, the relaxation shifts toward higher frequencies as *T* increases.

**Figure 5:**
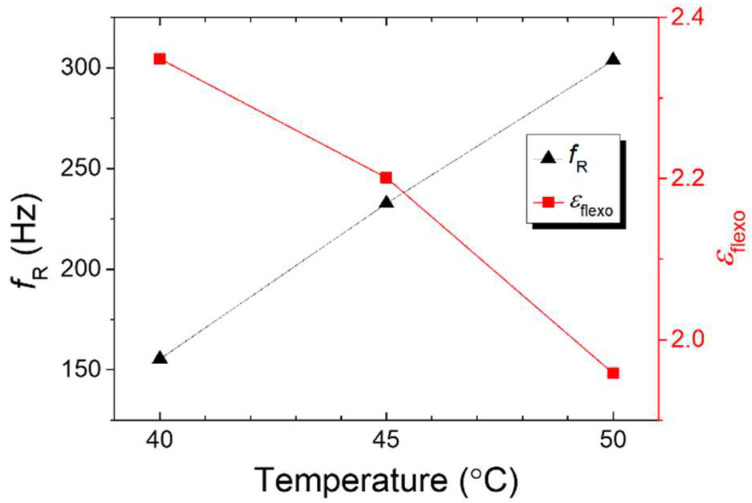
*T*-dependent relaxation frequency and flexoelectric polarization strength in the dye-free CB7CB/R5011 system.

Building on the detailed investigation of the relaxation behavior of the CB7CB/R5011 system, we applied Debye’s dielectric relaxation theory [32,33] to analyze and simulate the material’s response across a specific frequency range. The experimental results were fitted using the following equations:

(5) $$\varepsilon^{\prime }=\varepsilon_{H}+\frac{\left(\varepsilon_{L}-\varepsilon_{H}\right)}{1+f^{2}/f_{R}^{2}}$$

and

(6) $$\varepsilon_{\mathrm{flexo}}=\varepsilon_{L}-\varepsilon_{H},$$

yielding key parameters: high-frequency dielectric permittivity *ε*_H_, low-frequency dielectric permittivity (*ε*_L_), and dielectric relaxation frequency *f*_R_ at varying *T*, all derived from Equation (5). The relaxation strength of the flexoelectric polarization (*ε*_flexo_) was subsequently calculated using Equation (6). As illustrated in Figure 5, *ε*_flexo_ decreases with increasing *T*, whereas *f*_R_ shifts toward higher *f*, indicating *T*-dependent relaxation dynamics.

**Figure 6:**
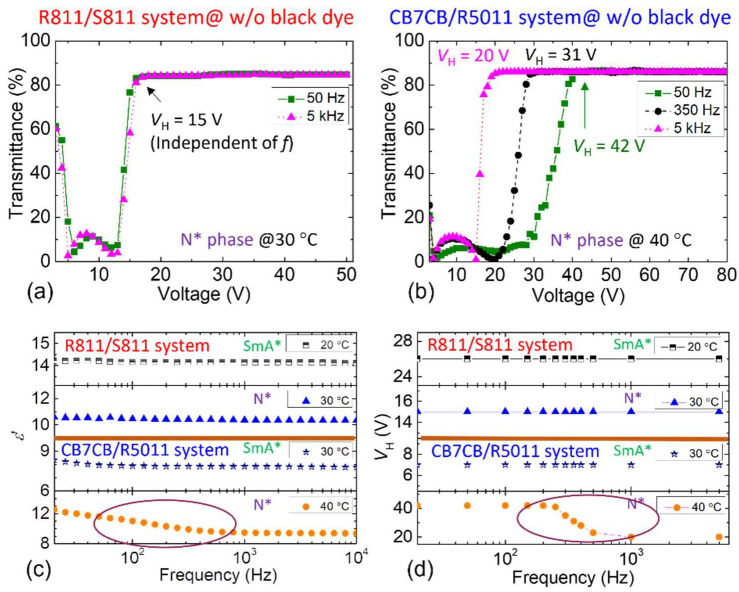
*V*–*T%* curves of (**a**) the R811/S811 and (**b**) CB7CB/R5011 systems at various frequencies. (**c**) Real-part dielectric spectra and (**d**) frequency-dependent *V*_H_ of SmA* and N* phases for the R811/S811 and CB7CB/R5011 systems. The nematic host of these CLC samples is E7.

To leverage switching between transparent (H) and scattering (FC) states, we examined *V*_H_ characteristics of the two material systems. As demonstrated in Figure 6a,b, the voltage–transmittance (*V*–*T*%) curves were measured for the R811/S811 and the CB7CB/R5011 systems in the N* phase, using AC voltage at 50 Hz and 5 kHz—representing the lower and upper limits of the human visual frame rate, respectively. For the R811/S811 system (Figure 6a), the *V*–*T* curves at both frequencies are nearly identical, indicating that *V*_H_ is frequency-independent. Conversely, the CB7CB/R5011 system exhibits frequency-dependent behavior, with *V*_H_ decreasing as frequency increases. Notably, *V*_H_ in the CB7CB/R5011 system is considerably higher than that of the R811/S811 control group. This suggests that the observed frequency dependence in the CB7CB/R5011 system is linked to dielectric relaxation induced by the flexoelectric polarization. Supporting this, Figure 6c shows pronounced relaxation only in the N* phase (40 °C) of the CB7CB/R5011 system. Figure 6d presents corresponding *V*_H_ measurements across frequencies, confirming that frequency dependence of *V*_H_ occurs exclusively in the N* phase, further validating its association with flexoelectric relaxation.

**Figure 7:**
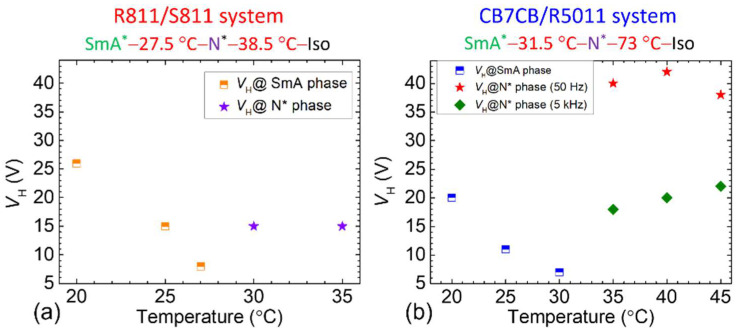
*V*_H_ as a function of *T* for the undoped (**a**) R811/S811 and (**b**) CB7CB/R5011 CLC systems. The nematic host of these CLC samples is E7.

Figure 7a,b present *V*_H_ variation with *T* for the R811/S811 and CB7CB/R5011 systems, respectively. In the R811/S811 system, *V*_H_ of 8 V_rms_ at 27 °C (near the SmA*–N* phase transition *T*) is notably lower than that at 30 °C (*V*_H_ = 15 V_rms_), likely due to changes in the order parameter. A similar trend is monitored in the CB7CB/R5011 system (Figure 7b). However, previous results indicate that *V*_H_ in the N* phase of the CB7CB/R5011 system is frequency-dependent. Thus, *V*_H_ was measured simultaneously at two frequencies: 50 Hz and 5 kHz. This study emphasizes the distinct *V*_H_ values near the SmA*–N* phase transition, attributed to order parameter variation, to permit switching between the SmA*–H and N*-FC states.

### 3.2. Contrast Ratio and Haze Values

**Figure 8:**
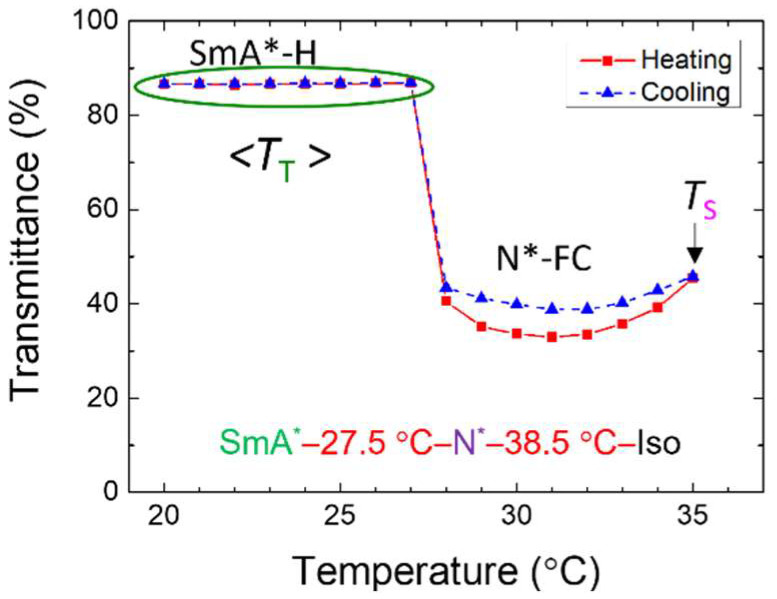
*T*-dependent average transmittance of the undoped R811/S811 system (without dye), with the phase transition sequence illustrated. The nematic host of these CLC samples is E7.

As shown in Figure 8, the transmission spectra of the SmA*–H and N*-FC states in the R811/S811 system (without black dye) were measured by a fiber-optic spectrometer under continuous 20-V_rms_, 5 kHz AC voltage. The relationship between average transmittance and *T* was plotted for the 400–700 nm wavelength range. *T* was gradually increased from 20 °C to 35 °C at a rate of 1 °C/min. Upon reaching *T* = 28 °C—above the SmA*–N* phase transition *T*, the sample transitioned from the transparent SmA*–H state to the scattering N*-FC state under fixed voltage conditions. *T* was then raised to 35 °C and subsequently lowered back to 20 °C at the same rate, as indicated by the blue line in Figure 8. When *T* drops below the SmA*–N* phase transition point and the voltage remain unchanged, the sample reverts from the scattering N*-FC state back to the initial transparent SmA*–H state. The heating and cooling curves show a hysteresis effect, and the transmittance of the N*-FC state varies with *T*. Tests confirm that this behavior is independent of waiting time during heating and cooling. In contrast, the transmittance in the SmA*–H state remains constant. The average total transmittance $\langle T_{t}\rangle$ across all *T* in the SmA* phase is calculated, and CR is defined as $\langle T_{t}\rangle$ divided by the transmittance of scattering state (*T*_s_), as shown in the equation

(7) $$\mathrm{CR}=\frac{\langle T_{t}\rangle }{T_{s}}.$$

This metric effectively evaluates device performance across accessible states. The same method is used for all subsequent CR calculations.

**Figure 9:**
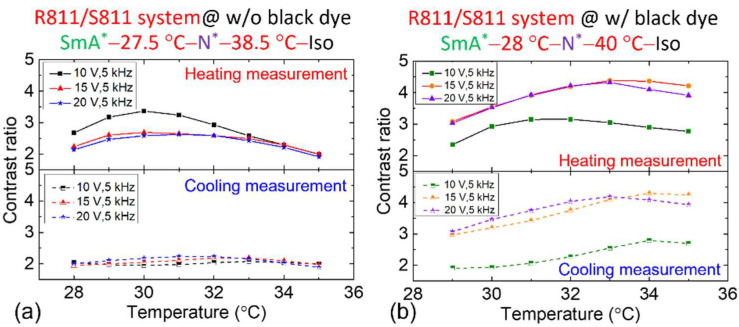
*T*-dependent CR for the R811& S811 system (**a**) without and (**b**) with black dye under various AC voltage amplitudes at 5 kHz during heating and cooling, with the phase transition sequences designated before and after doping black dye. The nematic host of these CLC samples is E7.

As displayed in Figure 9a, CR of the R811/S811 system without black dye varies with *T* under different voltages during heating and cooling, ranging between 2 and 3. Figure 9b illustrates the CR behavior after doping with black dye. With the dye, CR ranges from 2 to 3 under a 10 V_rms_ and 5 kHz, and increases to 3–4 under a 15 V_rms_ or 20 V_rms_—higher than that in the undoped sample. This suggests that black dye doping effectively enhanced CR. For instance, at 32 °C and 20 V_rms_, CR of the doped sample increased by 62%.

The black dye operates in a guest–host, dichroic manner: its transition dipole moment (i.e., absorption axis) aligns with the liquid-crystal director, representing the average molecular orientation. As a result, the effective optical absorption depends on the relative angle between the dye axis and the incident electric field. The contrast ratio (CR) enhancement enabled by the dye stems from this guest–host dichroism. In the SmA*–H state, the dye axis is nearly perpendicular to the optical field, leading to minimal effective absorption. In contrast, the N*-FC state exhibits increased orientational disorder, which enhances the projection of the field onto the dye axis. Combined with multiple scattering effects, this results in significantly stronger attenuation. Consequently, the transmittance in the FC state (denoted as *T*_FC_, representing the minimum) decreases more than in the H state (*T*_H_, representing the maximum), thereby increasing the contrast ratio, defined as CR ≡ *T*_max_/*T*_min_. Clearly, reducing the denominator is an effective strategy for boosting CR.

**Figure 10:**
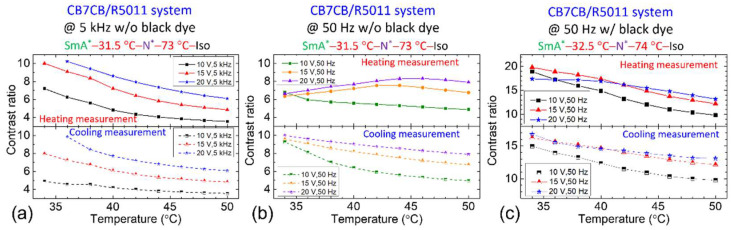
*T*-dependent CR for the CB7CB/R5011 system without black dye at various (**a**) 5 kHz and (**b**) 50 Hz voltages, and (**c**) for the dye-doped counterpart at 50 Hz AC voltages. The nematic host of these CLC samples is E7.

Figure 6 reveals an experimental correlation between the strength of the flexoelectric polarization in the CB7CB/R5011 system and the frequency of the applied AC voltage. To elucidate this relationship, measurements were conducted at two frequencies: 5 kHz and 50 Hz. CR was evaluated across varying voltage amplitudes in relation to *T*, with results presented in Figure 10, where CR was calculated as outlined in Equation (7). As shown in Figure 10a, CR increases significantly with rising voltage at a fixed frequency of 5 kHz. At 20 V_rms_ (blue line), CR values at lower *T* are unavailable due to the LC molecules approaching the H state at 34 °C. Notably, CR during heating and cooling spans from 7 to 10, indicating a markedly high performance than the R811/S811 system (Figure 9a). Figure 10b presents *T*-dependent CR at 50 Hz; unraveling a similar trend: CR increases with voltage amplitude. Under 20 V_rms_ at 50 Hz (violet line), CR during heating ranges from 7 to 8—slightly lower than at 5 kHz (Figure 10a)—while cooling measurements show a smoother variation between 8 and 10. Beyond CR, other factors merit consideration. Compared with 5 kHz, 50 Hz AC voltage is more economical and cost-effective. Thus, 50 Hz was selected to evaluate CR after doping the CB7CB/R5011 system with black dye. Figure 10c shows that at 20 V_rms_ (blue line), CR ranges from 15 to 17, confirming the superior optical performance of the doped CB7CB/R5011 system over the R811/S811 system. Consequently, subsequent experiments will focus exclusively on the CB7CB/R5011 system.

In addition to transmission spectra measurements, this study utilizes a haze meter with a *T* controller to evaluate haze. As defined in Equation (4), the total transmittance *T*_t_ and diffuse transmittance *T*_d_ from Table 3 and Table 4 can be substituted into *I*_t_ and *I*_d_ in the equation. Based on the results in Figure 10, a voltage of 20 V_rms_ at 50 Hz was selected as the applied condition for haze measurements. Moreover, since heating and cooling exhibited similar trends, haze was measured exclusively during the heating process. Table 3 details the *T*-dependent haze behavior of the CB7CB/R5011 system without black dye. Under continuous application of 20-Vrms, 50 Hz voltage in the SmA* phase, the samples remained in the H state, achieving *T*_t_ up to 89% and a minimal haze of approximately 1%. Notably, this 1% originates from the ITO-coated glass substrates: the ITO films exhibit finite surface micro-roughness and grain boundaries; moreover, if the measurement footprint overlaps patterned-ITO steps/edges, the associated thickness discontinuity introduces a minute additional scattering component. These mechanisms are forward scattering and, by the integrating-sphere definition (scattering angle > 2.5°), are counted as diffuse transmittance; hence, a residual approximately 1% haze may appear even in the H state. When *T* exceeds the SmA*–N* phase transition point under the same voltage, the samples transitioned from the transparent SmA*–H state to the scattering N*-FC state, with haze increasing to 72–74%. Table 4 presents the data samples doped with black dye. In the H state, *T*_t_ decreased to approximately 64% due to absorption by the dye, while haze slightly increased to around 1.5%. Regarding this 1.5% haze value, although it is marginally higher than the almost 1% observed for the undyed CB7CB/R5011 cells (H state), the increase does not arise from enhanced scattering; rather, the denominator *T*_t_ is reduced by absorption while *T*_d_ remains nearly 1%—dominated by weak forward scattering from the ITO film—thereby increasing the *T*_d_/*T*_t_ ratio. Upon transitioning to the N*-FC state, haze rose to between 77 and 79%. These findings indicate that black dye enhances both CR and haze. However, continuous application of AC voltage is essential to maintain the SmA*–H state with high transmission. The following subsection will focus on refining the experimental setup to reduce energy consumption and improve electro-optical performance.

### 3.3. Improvement and Optimization of Experimental Framework

The continuous-voltage mode offers simplicity in circuit design. In contrast, our experimental setup (Figure 2) was adapted for variable-voltage operation. A threshold *T* in the N* phase was defined as the default setting. A small continuous voltage was applied, and adjusted dynamically: when *T* exceeded the threshold, the voltage was withdrawn; when *T* fell below it, the voltage increased by 1 V_rms_ per 1 °C drop. Applying a subthreshold voltage in the N* phase yielded a scattering state with reduced transmittance. Additionally, the sample exhibited lower *V*_H_ as *T* approached the SmA*–N* phase transition. In the N*-FC state, the variable-voltage mode produced similar low transmittance as the continuous-voltage mode but consumed less energy (Figure 2), making it more energy-efficient.

**Figure 11:**
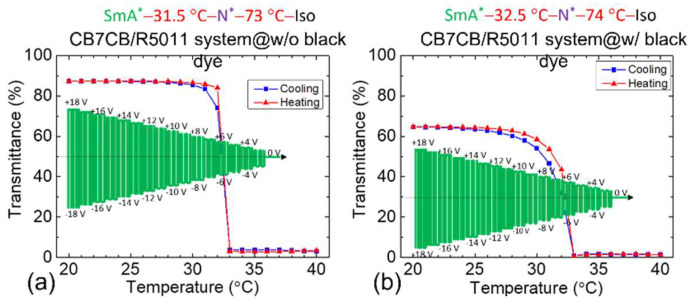
*T*-dependent transmittance in the variable-voltage mode for the CB7CB/R5011 samples (**a**) without black dye and (**b**) with black dye. The nematic host of these CLC samples is E7.

Figure 11a shows transmittance vs. *T* for samples without black dye under variable-voltage control. Measurements were performed during cooling (blue curve) and then heating (red curve). Initially, *T* was held at 40 °C, then decreased at 1 °C·min^−1^. At 35 °C, a 3-V_rms_, 50 Hz AC voltage was applied, increasing by 1 V_rms_ per 1 °C drop—reaching 18 V_rms_ at 20 °C. This progression is illustrated in the green pattern of Figure 11a. As *T* decreased and approached the SmA*–N* transition, the sample shifted from low-transmission N*-FC state to high-transparent SmA*–H state. Tests confirmed that the LC in the N* phase was not fully driven under variable-voltage conditions, resulting in low transmittance, whereas the SmA* phase maintained high transmittance. During heating, *T* was elevated from 20 °C to 40 °C at the same rate (red curve), using the same voltage profile. The heating and cooling curves showed consistent trends. Figure 11b presents *T*-dependent transmittance for black dye-doped samples under the same conditions. As *T* neared the SmA*–N* phase transition *T*, transmittance changes diminished (with increasing *T*), indicating a gray-scale effect. The N* phase retained low transmittance.

We then measured haze for samples without and with black dye under the variable-voltage mode. Table 5 shows that haze in the transparent SmA*–H state ranged from 1.1 to 1.3. Upon entering the SmA*–N* phase transition and transitioning to the scattering N*-FC state, haze increased to 81–82%, significantly higher than in the continuous-voltage mode (Table 3). Table 6 details haze variation with *T* for dye-doped samples. These CB7CB/R5011 samples also showed higher haze in the scattering state compared with the continuous-voltage mode. It is notable that haze reached 88% as *T* exceeded the SmA*–N* phase transition point, surpassing values in Table 4. Overall, variable-voltage mode produced higher haze than in continuous-voltage mode, with dye-doped samples exhibiting slightly greater haze than undoped ones.

Note that, in Table 6 (dye-doped cells under the varying-voltage protocol), the gradual rise in the diffuse component *T*_d_—manifested as an H-state haze increase from nearly 1.5% to 8.5%—is attributed to pretransitional optical scattering near the SmA*–N* boundary. As temperature approaches the transition point, the smectic layer’s long-range order weakens and director fluctuations with incipient focal-conic nucleation emerge, which enhance small-angle forward scattering even while the sample is still macroscopically in the H state. This effect is amplified by the driving protocol: in the varying-voltage mode the applied voltage is gradually reduced during heating, so the electric torque becomes insufficient to fully sustain a perfectly homeotropic configuration close to the transition; the reduced field margin leads to additional low-angle scattering (higher *T*_d_). Furthermore, in the dye-doped system the guest–host mixture exhibits a slightly lower effective order parameter at elevated temperatures and a moderate temperature-dependent absorption, both of which make pretransitional fluctuations more visible in *T*_d_. Consequently, *T*_d_ (and thus haze) increases continuously from almost 1.5% to 8.5%. By contrast, Table 5 (dye-free cells) shows nearly constant *T*_d_ (approximately 1%) because (i) no dye-induced absorption or order-parameter reduction is present, and (ii) under the same protocol the field margin above *V*_H_ remains larger across the examined temperature window, so pretransitional scattering remains negligible.

## 4. Conclusions

This study investigated two LC systems—R811/S811 and CB7CB/R5011—both capable of inducing the SmA* phase and enabling passive, reversible switching between the transparent SmA*–H and scattering N*-FC states without surface orientation treatment. These findings present a promising strategy for LC-based smart windows and optical switching devices.

To enhance electro-optical performance and reduce energy consumption, the experimental architecture was further optimized. Comparative analysis revealed that the CB7CB/R5011 system offers a significantly broader Δ*T*_N*_ than R811/S811 (Figure 3), providing a wider operating window suitable for practical applications. The addition of black dye improved optical properties in both systems without altering the phase transition *T*, as confirmed by optical texture and dielectric measurements (Figure 3).

Incorporating the bent-core LC dimer CB7CB introduced pronounced flexoelectric polarization in the CB7Cb/R5011 system, resulting in frequency-dependent *V*_H_ behavior in the N* phase (Figure 4, Figure 5 and Figure 6). Electro-optical performance was evaluated using average transmission (400–700 nm) and CR calculations (Equation (7)). As shown in Figure 9, the R811/S811 system exhibited CR values of 2–3 (undoped) and 3–4 (doped) under continuous-voltage mode at a voltage of 20 V_rms_. Concurrently, the CB7CB/R5011 system achieved CR values of 7–10 (undoped) and 15–17 (doped) (Figure 10), indicating superior performance and enhanced CR with black dye incorporation.

Haze measurements further supported the scattering efficacy of the CB7CB/R5011 system. Under continuous-voltage mode, haze value reached 73% (undoped) and 78% (doped) (Table 3 and Table 4, respectively). These high haze values are indicative of satisfactory performance as a scattering device. Upon transitioning to a variable-voltage mode, haze increased to 81% and 88%, respectively (Table 5 and Table 6), while energy consumption was reduced. These results manifest that the variable-voltage mode not only improves energy efficiency but also enhances electro-optical properties. The novel switching mechanism and variable-voltage strategy introduced in this work offer substantial potential for future development. This flexible voltage scheme may enable broader applications in smart windows, optical switches, and adaptive window blinds.

## Figures and Tables

**Table 1:** Formulation of two CLC variants containing R811/S811 doped in the nematic host E7.

Sample	E7 (wt.%)	R811 (wt.%)	S811 (wt.%)	S428 (wt.%)
S1-1	70.0	20.0	10.0	0.0
S1-2	69.3	19.8	9.9	1.0

**Table 2:** Composition of two CLC samples incorporating CB7CB/R5011 doped in the nematic host E7.

Sample	E7 (wt.%)	CB7CB (wt.%)	R5011 (wt.%)	S428 (wt.%)
S2-1	49.5	49.5	1.0	0.0
S2-2	49.0	49.0	1.0	1.0

**Table 3:** Haze measurement results for the CB7CB/R5011 system without black dye doped in the nematic host E7.

Temperature (°C)	*T*_t_ (%)	*T*_d_ (%)	Haze (%)
24	89.23	0.93	1.05
26	89.23	0.93	1.05
28	89.23	0.92	1.04
30	89.24	0.97	1.04
32	89.24	0.94	1.05
34	77.08	57.49	74.58
36	77.07	57.30	74.34
38	77.06	57.09	74.09
40	77.11	56.53	73.31
42	77.14	56.14	72.78
44	77.17	55.88	72.42

**Table 4:** Haze measurement results for the CB7CB/R5011 system with black dye doped in the nematic host E7.

Temperature (°C)	*T*_t_ (%)	*T*_d_ (%)	Haze (%)
24	64.35	0.98	1.54
26	64.36	0.98	1.55
28	64.36	1.00	1.54
30	64.35	0.99	1.54
32	64.35	0.98	1.54
34	30.42	24.30	79.86
36	31.18	24.55	78.72
38	31.19	24.47	78.45
40	31.16	24.40	78.29
42	31.15	24.35	78.19
44	31.18	24.29	77.90

**Table 5:** Measured haze values for the CB7CB/R5011 system without black dye doped in the nematic host E7 under variable-voltage operation.

Temperature (°C)	*T*_t_ (%)	*T*_d_ (%)	Haze (%)
24	89.25	1.06	1.18
26	89.27	1.07	1.19
28	89.23	1.07	1.21
30	89.21	1.08	1.21
32	89.13	1.19	1.34
34	77.16	63.84	82.74
36	77.54	63.69	82.14
38	77.67	63.57	81.85
40	77.32	63.34	81.92
42	77.35	62.96	81.40
44	78.87	63.98	81.12

**Table 6:** Measured haze values for the CB7CB/R5011 system with black dye doped in the nematic host E7 under variable-voltage operation.

Temperature (°C)	*T*_t_ (%)	*T*_d_ (%)	Haze (%)
24	64.07	1.45	2.26
26	63.33	2.47	3.90
28	61.39	4.65	7.57
30	59.56	6.37	10.70
32	54.42	8.47	15.59
34	55.13	12.23	22.18
36	47.52	17.31	36.42
38	35.33	23.29	65.93
40	21.67	19.36	89.31
42	21.64	19.39	89.61
44	21.62	19.35	89.51

## Data Availability

The original contributions presented in this study are included in the article. Further inquiries can be directed to the corresponding author.
